# An SOI-Structured Piezoresistive Differential Pressure Sensor with High Performance

**DOI:** 10.3390/mi13122250

**Published:** 2022-12-17

**Authors:** Zebin Xu, Jiahui Yan, Meilin Ji, Yongxin Zhou, Dandan Wang, Yuanzhi Wang, Zhihong Mai, Xuefeng Zhao, Tianxiang Nan, Guozhong Xing, Songsong Zhang

**Affiliations:** 1 School of Microelectronics, Shanghai University, Shanghai 201800, China; 2 JiuFengShan Laboratory, Future Science and Technology City, Wuhan 420000, China; 3 Shanghai Industrial μTechnology Research Institute, Shanghai 201899, China; 4 Institute of Microelectronics, Chinese Academy of Sciences, Beijing 100029, China; 5 Institute of Microelectronis, Tsinghua University, Beijing 100084, China

**Keywords:** high-sensitivity, low temperature drift, piezoresistive effect, silicon-on-insulator (SOI) pressure sensor, microelectromechanical systems (MEMS)

## Abstract

This paper presents a piezoresistive differential pressure sensor based on a silicon-on-insulator (SOI) structure for low pressure detection from 0 to 30 kPa. In the design phase, the stress distribution on the sensing membrane surface is simulated, and the doping concentration and geometry of the piezoresistor are evaluated. By optimizing the process, the realization of the pressure sensing diaphragm with a controllable thickness is achieved, and good ohmic contact is ensured. To obtain higher sensitivity and high temperature stability, an SOI structure with a 1.5 µm ultra-thin monocrystalline silicon layer is used in device manufacturing. The device diaphragm size is 700 µm × 700 µm × 2.1 µm. The experimental results show that the fabricated piezoresistive pressure sensor has a high sensitivity of 2.255 mV/V/kPa and a sensing resolution of less than 100 Pa at room temperature. The sensor has a temperature coefficient of sensitivity (TCS) of −0.221 %FS/°C and a temperature coefficient of offset (TCO) of −0.209 %FS/°C at operating temperatures ranging from 20 °C to 160 °C. The reported piezoresistive microelectromechanical systems (MEMS) pressure sensors are fabricated on 8-inch wafers using standard CMOS-compatible processes, which provides a volume solution for embedded integrated precision detection applications of air pressure, offering better insights for high-temperature and miniaturized low-pressure sensor research.

## 1. Introduction

With the development of microelectromechanical manufacturing technology, the microelectromechanical systems (MEMS) pressure sensor has shown excellent performance in air pressure monitoring and liquid measurement [1,2,3]. High-performance pressure sensors have attracted more and more attention and are widely used in consumer electronics, the mechanical industry, aerospace, biomedical, and other fields. [4]. Various sensing principles, including piezoresistive, capacitive, piezoelectric, optical, and resonant sensing, have been adopted in different types of pressure sensors [5,6]. In contrast, piezoresistive pressure sensors have remarkable characteristics such as low output impedance, stronger resistance against electromagnetic noise, low power consumption, a wide detection range, and a simple manufacturing process [7,8]. To meet the demands of their applications, many automotive industries and consumer electronics now require high-precision, miniaturized, high-temperature-resistant pressure sensors [9]. Therefore, the development of miniaturized high-temperature MEMS piezoresistive pressure sensors has become one of the key research directions [10]. In research, SOI technology has been developed to fabricate piezoresistive pressure sensors with good high-temperature adaptability. This technology breaks the high-temperature application limitation of the conventional block-silicon-based piezoresistive pressure sensors and enables the pressure sensors to be applied to harsh environments at 250 °C and even higher temperatures. [11,12,13,14,15,16]. In addition, SOI pressure sensors can be well controlled in the batch manufacturing process compared with some new materials such as silicon carbide, silicon nanowires, and graphene [17,18,19,20,21]. Li, S. et al. proposed an SI pressure sensor with a sensitive diaphragm width of 1000 µm, which was realized for use at 350 °C [22]. Li, C. et al. fabricated a four-grooved rood beam SOI piezoresistive pressure sensor with a high sensitivity for low-pressure measurements at 150 °C, but its sensing diaphragm size could reach 3.6 mm× 3.6 mm × 0.03 mm [23]. Meng, Q. et al. proposed a piezoresistive pressure sensor. Through numerical simulation optimization, the piezoresistor position was arranged in the center and edge of the diaphragm, and the piezoresistive thickness was 2 µm. The characterization results showed that the sensitivity was 37.79 mV/V/MPa, the hysteresis of 0.09%FS was low, and repeatability was 0.03%FS, but the sensing structure dimension was as large as 5 mm × 5 mm × 0.9 mm [24]. Balavalad, K.B. et al. designed a miniature piezoresistive SOI pressure sensor with the most temperature compensation using a dual Wheatstone bridge, which ultimately provided a sensitivity of 298 mV/MPa [25]. Yao, Z., et al. investigated a high-temperature SOI piezoresistive pressure sensor with integrated signal conditioning circuitry for long-term operation in the range of 50 °C to 220 °C, but the sensitivity of the device was only 0.42 mV/V/KPa [11]. Gao et al. designed a C-structure SOI piezoresistive pressure sensor with a wide pressure range of 0–45 bar on the sensor chip, but the output sensitivity was only 9.21 mV/bar [26]. Song, Zi, et al. designed an SOI pressure sensor with a Wheatstone bridge on the lower surface of the pressure diaphragm to avoid contact with the external environment, which had a wide pressure range, but the sensitivity of the device was 20 mV/V/KPa [27]. Sensor companies such as Goodrich, Gefran, and Kulite have also introduced SOI high-temperature pressure sensors for medium- and high-temperature environments [28,29]. Enhancing the high-temperature characteristics of the sensor while achieving high accuracy is an issue to be considered. On the other hand, some researchers have developed diaphragm structures to improve the performance of sensors [30,31]. However, most sensors have large sensing diaphragms and low combined accuracy. As mentioned earlier, a great deal of research on MEMS piezoresistive pressure sensors has focused on achieving improved sensor sensitivity and high-temperature suitability. However, the balance between a smaller footprint and excellent sensing performance of high-temperature pressure sensors needs to be further explored.

In this work, an SOI-based piezoresistive differential pressure sensor with high performance was designed for air pressure monitoring from 0 to 30 kPa. The sensing structure was optimized by simulating the stress distribution parameters of the silicon diaphragm at different pressures, equalizing the size and thickness of the diaphragm to achieve highly concentrated stress, and controlling the process parameters (such as the dose of ion implantation and exposure of lithography) well during the fabrication process to meet the target resistance value and position of the piezoresistive. The characteristics, such as sensitivity and temperature drift, of the devices were extracted by static and dynamic environmental tests, and the feasibility of realizing multilevel performance devices with high sensitivity, small size, and high-temperature resistance was determined.

## 2. Principle and Design

### 2.1. Piezoresistive Effect

Sensitivity, repeatability, and temperature drift characteristics are particularly important performance indicators of piezoresistive pressure sensors [32]. A thin-film structure is often used to sensitively detect the changes in external pressure [33]. The SOI piezoresistive pressure sensor we developed is based on the piezoresistive effect, and the resistance has the characteristic of changing with pressure [34,35,36,37,38]. The resistance to change is expressed as:

(1) $$\frac{\Delta R}{R}=\pi_{l}\sigma_{l}+\pi_{t}\sigma_{t}$$

where *π_l_* and *π_t_* are longitudinal and transverse piezoresistive coefficients, respectively; *σ_l_* and *σ_t_* are longitudinal and transverse stresses, respectively.

**Figure 1:**
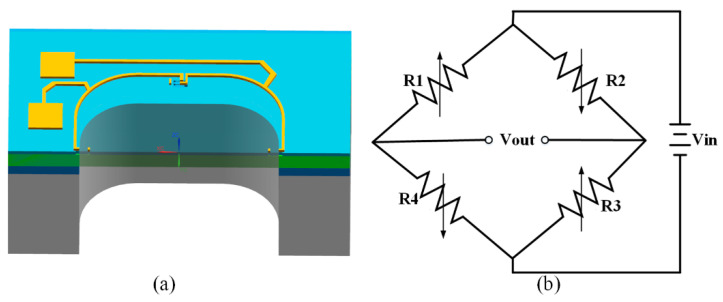
(**a**) Half-section view of the physical model; (**b**) Wheatstone bridge.

The piezoresistive pressure sensor we developed is based on an SOI wafer with piezoresistors configured in a Wheatstone bridge connection on the top layer of P-type monocrystalline silicon, as shown in Figure 1.

According to the Wheatstone bridge principle, the output voltage is given by Equation (2), and sensitivity is given by Equation (3).

(2) $$V_{\mathrm{out}}=V_{in}\frac{\Delta R}{R}$$

(3) $$S=\frac{\Delta V}{P_{I}-P_{M}}.\frac{1}{V_{in}}=\frac{\Delta V}{\Delta P\cdot V_{in}}$$

where *V_in_* is the excitation voltage; Δ*V* is the full-scale output, representing the change in the output voltage of the device from pressure *P_I_* to *P_M_*.

### 2.2. Sensor Design

The sensitivity of the sensor largely depends on the size of the diaphragm and piezoresistive as well as the position of the piezoresistor with respect to the location of the fixed anchor of the sensing structure. In the working range below the burst pressure, the sensitivity of a particular sensor depends on the amount of deflection of the diaphragm [13]. To exert the maximum piezoresistive effect of the device diaphragm and achieve higher sensitivity, these parameters need to be properly designed. Piezoresistors are usually laid out in the high-stress distribution region of the diaphragm to improve the sensitivity of the device [39,40,41,42].

**Figure 2:**
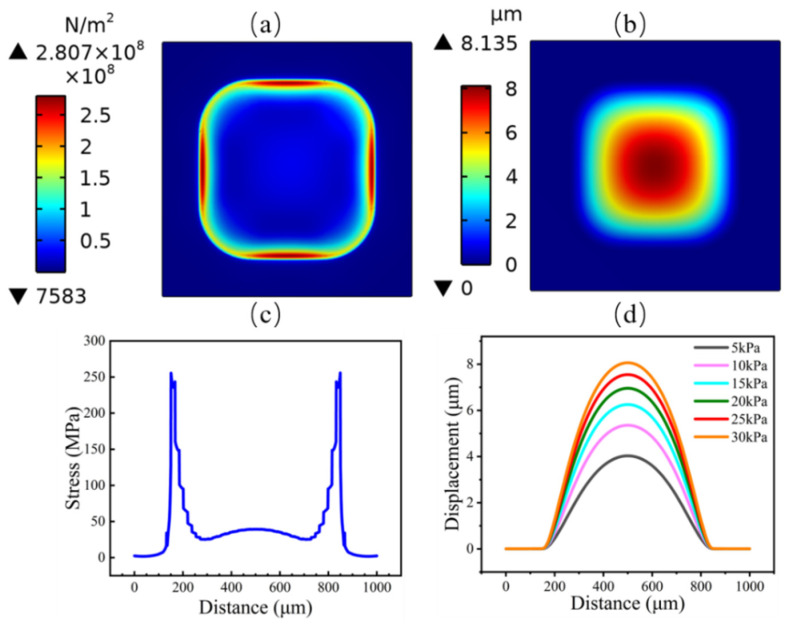
Stress and displacement distribution on a square diaphragm with a side length of 700 µm: (**a**) stress of the diaphragm; (**b**) displacement of the diaphragm; (**c**) variation in the stress component along the diaphragm centerline; (**d**) variation in the displacement along the diaphragm centerline.

The sensors were designed for a range of 0–30 kPa with a size of 700 µm × 700 µm × 2.1 µm. The physical model (as in Figure 1) was used to estimate the diaphragm displacement, and the von Mises stress distribution and the simulation results are shown in Figure 2.

A pressure of 30 kPa was applied to the diaphragm. The maximum stress was symmetrically distributed in the center of the edge of the diaphragm, as shown in Figure 2a. The maximum stress location can be seen in Figure 2c within 15–20 µm from the edge of the diaphragm, which provides design support for the location of the piezoresistor. The displacement of the diaphragm is depicted in Figure 2b. Different displacement values could be observed for the diaphragm under different pressures, with the largest displacement occurring in the center of the diaphragm, as shown in Figure 2d. The simulation parameters and results for the diaphragms are shown in Table 1.

According to the stress simulation results of diaphragms, it was determined that the stress at the center of the film edge was the largest, and the piezoresistor should be placed in this position.

In order to position the piezoresistor in the high-stress region of the diaphragm, the size and number of turns of the piezoresistor need to be considered. If the piezoresistor size is too large, the sensitivity of the sensor will be reduced after the stress is averaged. The piezoresistor was downsized and designed with a specific number of turns.

**Figure 3:**
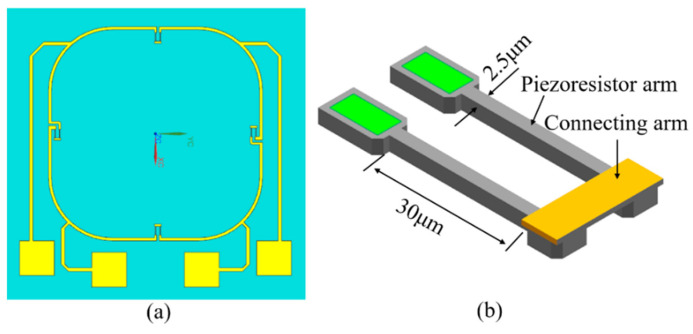
Piezoresistive dimensional design: (**a**) configuration of the sensing membrane and the layout of interconnection; (**b**) schematic of one piezoresistors.

As shown in Figure 3, a curved piezoresistor with two turns was selected, and the entire part consisted of a piezoresistor with a planar dimension of approximately 2.5 µm × 30 µm and a connecting arm. Among them, the heavily doped region was connected to the piezoresistors and the outer metal electrode of the diaphragm because the piezoresistive effect decreases with the increase in the doping concentration, and this region has a small piezoresistive effect to avoid excessive extra resistance.

## 3. Manufacturing Process

The two most critical processes in the manufacturing of our piezoresistive devices are ion implantation and high-tempreature annealing. To form the shallow junction device, we need to precisely control the ion concentration dose and the annealing temperature. First, we simulated the doping junction depth size by setting two different energy pairs and determining the corresponding annealing temperature.

The piezoresistor was doped with impurity source boron ions at a fixed position. The doping dose was 2 × 10^14^ ions/cm^2^. Ion activation was performed by annealing at a temperature of 1000 °C for 15 min. The heavy doping dose was 2 × 10^15^ ions/cm^2^, and the doping energy was 20 keV. Ion activation was also performed by annealing at 1000 °C for 15 min. The formation of amorphization by depositing a barrier layer of silicon dioxide reduces the problem of ion channeling accompanying ion implantation [43,44].

**Figure 4:**
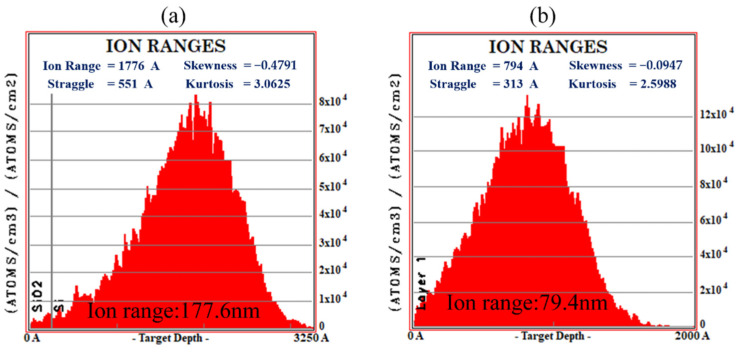
Ion implantation simulation and homogeneity experiments: (**a**) injection junction depth of 50 keV; (**b**) injection junction depth of 20 keV.

To minimize the ion channel effect, the thickness of the barrier layer was set to 25 nm, and the ion implantation was simulated using SRIM software (Dr. James F. Ziegler of Yale University, USA, developed the software). The simulation results are shown in Figure 4a, with the junction depths being 177.6 nm for ion injection energies of 50 keV. The heavy doping during device fabrication was simulated, and the results are shown in Figure 4b for 79.4 nm. The actual doping region of the resistance was determined and served as a reference for the optimization of the subsequent process to form a good ohmic contact, which could facilitate the electrical signal input and output during device operation.

The consistency of the sensor is an important indicator for the mass production of piezoresistive pressure sensors. First, it can be discussed based on the ion implantation process parameters. The ion implantation doping concentration *N*(*x*) is a function of the implanted beam current *I* and time *T*. When the implantation dose *Q* is fixed, the larger the beam current, the shorter the time [45]. It can be explained by the following formula:

(4) $$N\left(x\right)=\frac{Q}{\sqrt{2\pi }\Delta R_{P}}\left[-\frac{1}{2}\left(\frac{x-R_{P}}{\Delta R_{P}}\right)\right]$$

(5) $$Q=\frac{IT}{Ane}$$

where *e* is the electron charge, *n* is the ion charge, *A* is the injection area, *R_P_* is the projected range, and Δ*R_P_* is the standard deviation.

**Figure 5:**
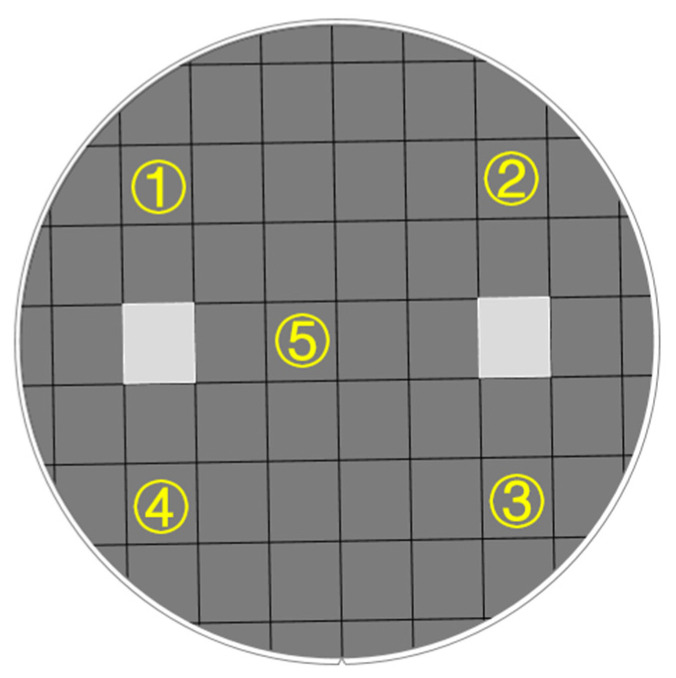
Uniformity test spot map on 8-inch wafers. ① to ⑤ are the selected test points.

The selection of the beam value is the key factor affecting the uniformity of injection. The overcurrent of the implanted beam leads to the accumulation of charge on the wafer, which affects the uniformity of the implantation. Therefore, it is necessary to set the optimal parameters according to the existing equipment and process capacity. Here, we set the beam to between 2 and 5 mA. After ion implantation and annealing, resistance measurements were performed at five points selected from a wafer with a diameter of 200 ± 0.2 mm (Figure 5), and the values are shown in Table 2 below. It can be seen that the uniformity of resistance was relatively high when the beam was 2 mA and 4 mA; the homogeneity obtained using the mean squared deviation calculation was 87% and 95.3%, respectively. At a certain dose, the processing time decreased with the increase in the beam current. Taking the process into account, a beam current of 4 mA was chosen as the reference value. Meanwhile, the subsequent process was controlled to improve the uniformity of the piezoresistance in general.

**Figure 6:**
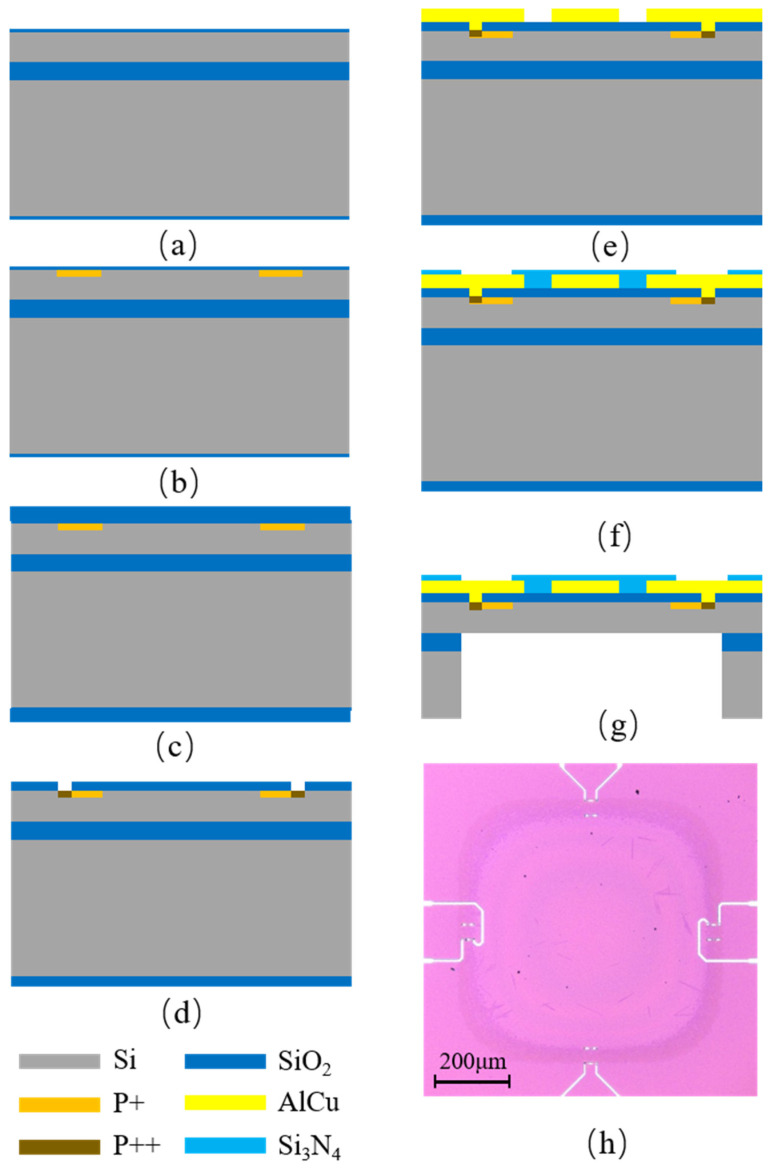
Fabrication process: (**a**) thermal oxidation; (**b**) boron light dope; (**c**) deposition of SiO_2_; (**d**) boron heavy dope; (**e**) metal wiring; (**f**) deposition of Si_3_N_4_; (**g**) back cavity release; (**h**) device sample.

We started the fabrication on an 8-inch SOI wafer, and the P-type doped Si device layer was used to suppress the leakage current. According to the wafer manufacturer, the device layer thickness was 1.5 ± 0.05 μm (resistivity: 8.5 to 11.5 Ωcm), and the buried oxide layer (BOX) thickness was 1 ± 0.025 μm. The specific manufacturing process is shown in Figure 6.

In the first step, we grew a 25 nm SiO_2_ layer onto the SOI wafer shown in Figure 6a to suppress ion channel effects. Boron ions were implanted into the top Si layer with a doping dose of 2 × 10^14^ ions/cm^2^ and a doping energy of 50 keV, then annealing in a high-temperature furnace tube, as demonstrated in Figure 6b. Then, a 0.4 µm SiO_2_ thin film layer was deposited using a low-pressure chemical vapor deposition (LPCVD) process, as shown in in Figure 6c. Photolithography and etching processes were used to expose heavily doped patterned regions. A 10 nm layer of SiO_2_ was grown by thermal oxidation and removed by buffered oxide etchant (BOE). SiO_2_ was then used as a hard mask for the heavy doping of boron ions. The doping dose was 2 × 10^15^ ions/cm^2^, and the ion implantation energy was 20 keV; it was also annealed in a high-temperature furnace tube in, as demonstrated in Figure 6d. To create the Wheatstone bridge electrode connection regions and wiring distribution, a 0.7 µm AlCu metal layer was deposited on top of the membrane and patterned using a photolithographic process, followed by an alloying process and an etching process, as shown in Figure 6e. A 0.2 µm Si_3_N_4_ thin film layer was deposited using a plasma-enhanced chemical vapor deposition (PECVD) process, followed by photolithography and etching processes to fabricate the electrode PAD portion, as shown in Figure 6f. After, the wafer grinding backside deep silicon etching process was performed with 1 µm BOX as an etching stopper. The final oxide dry etching process removed the BOX and released the cavity diaphragm structure, as shown in Figure 6g, and the optical image of a released device is shown in Figure 6h.

**Figure 7:**
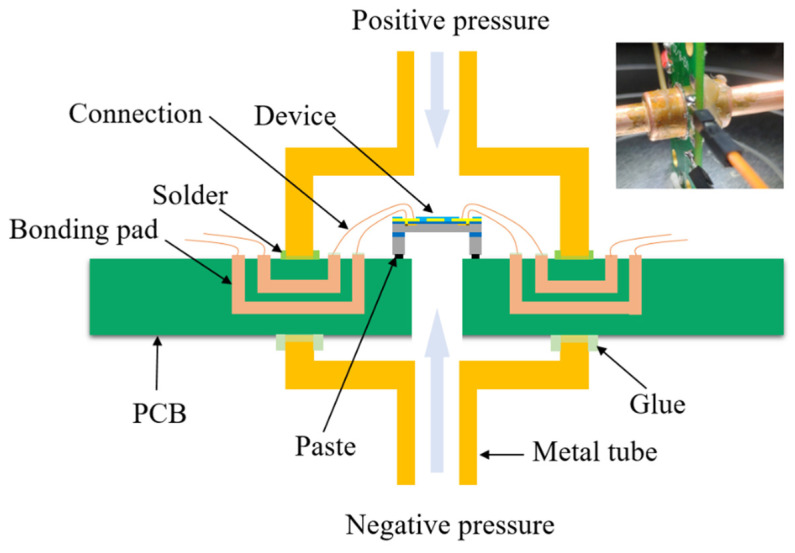
Schematic diagram of the process of packaging sensors.

Packaging is a well-known source of influence on the output stability of sensors. In order to form gas flow pathways and obtain sensor characteristics, the fabricated piezo-resistive differential pressure sensor chip was encapsulated, as shown in Figure 7. Existing sealing materials and adhesives were selected to increase the reliability of the sensor and the sealing and insulation of the package structure. The sensor chip was immobilized to a printed circuit board (PCB) with through holes using DELO BS3770 adhesive, which had a tensile strength of 2 MPa, provided good adhesion, and offered good temperature resistance and low packaging stress. The electrode pads on the chip and the pads on the PCB were connected by a wire bonding process. The electrodes were insulated and disconnected from each other. Metal tubes were also used to protect the sensor chip and served as inlet pressure ports. Through pressure testing, sealing metal tubes with soldering and insulating glue proved to be very effective. The wires that served as electrical connections were pulled out to apply input bias to the Wheatstone bridge and measure the sensor output.

## 4. Results and Discussion

**Figure 8:**
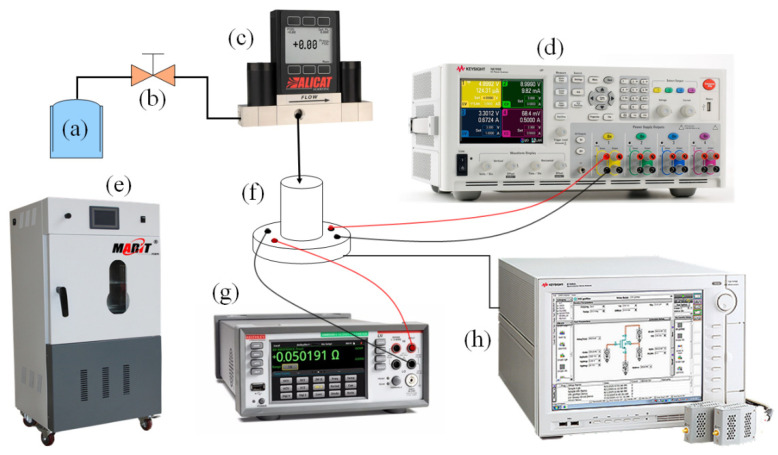
Test system: (**a**) gas pressure source; (**b**) gas intake switch; (**c**) pressure controller; (**d**) DC power analyzer; (**e**) vacuum drying oven; (**f**) test sample; (**g**) digital multimeter; (**h**) semiconductor device analyzer.

To measure the performance of sensors, the experimental setup shown in Figure 8 was used. The resistance value of the sensors was measured with a semiconductor analyzer through a probe station.

The compressed air pressure was generated by a pressure pump and controlled by a pressure valve. The packaged sensor was connected to the gas line via a rubber tube and was sealed by a fastening element. A flow meter was placed between the air pressure source and the device under test (DUT). The flow meter regulated the air pressure with precise control, which was advantageous when testing the pressure resolution of the sensor. A high current switching direct current (DC) power analyzer was used to provide a constant bias voltage of 1 V to the sensor’s Wheatstone bridge. The compressed air was applied through a pipe to the DUT at room temperature with a pressure range of 0–30 kPa. This pressure range can accommodate many applications such as consumer electronics (e.g., e-cigarettes and wearable electronics), medical (e.g., respirators), automotive electronics (e.g., engines), and industrial controls (e.g., high-temperature ovens).

A change in the output voltage value of the digital multimeter was observed and recorded to calculate the sensitivity and linearity of the available sensor. The repeatability error and hysteresis error of the sensor could be clearly seen by repeated measurements of the sensor and the results of the forward and reverse strokes. To measure the temperature drift characteristics of the sensor, the device was placed in a vacuum drying oven, and we set eight test temperature points in the range of 20 °C to 160 °C in steps of 20 °C, taking into account the ability of the test equipment to raise and lower the temperature and the comprehensive conditions of the device package. The output voltage in the full pressure range was measured in steps of 5 kPa, and the voltage output values with respect to the pressure changes at different temperatures were recorded with a multimeter. Finally, the TCO and temperature coefficient of sensitivity (TCS) of the sensor was calculated. 

Performance indicators such as sensitivity, repeatability, hysteresis, and zero-point temperature drift were the criteria used to evaluate the performance of the sensor. The characterization results of the sensor are plotted in the following.

**Figure 9:**
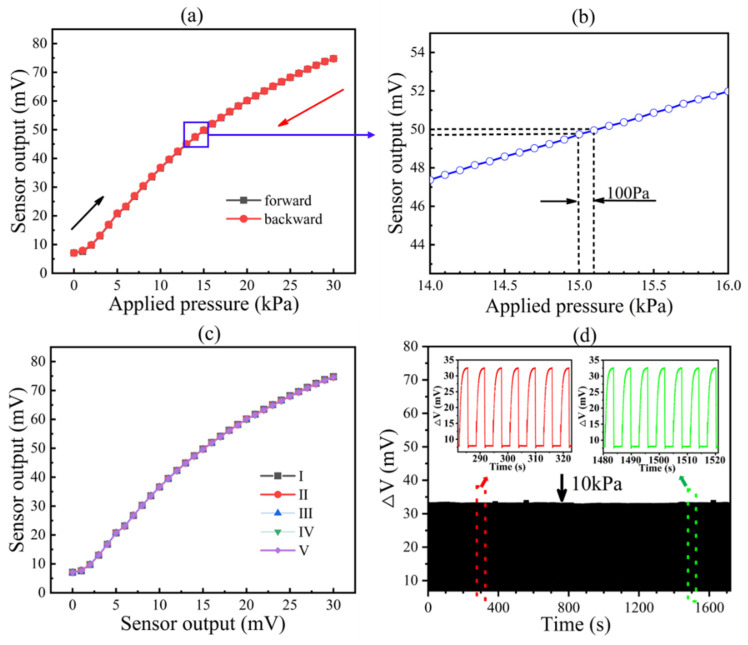
Sensor performance test results: (**a**) sensitivity and hysteresis; (**b**) real-time transient output by applying 100 Pa; (**c**) repeatability; (**d**) time response at 0 kPa to 10 kPa.

The performance of the fabricated piezoresistive pressure sensor subjected to pressure load at room temperature was tested. As shown in Figure 9a, the output voltage increased with applied pressure from 0 to 30 kPa under the influence of a 1 mV excitation voltage. The measurement results showed that the full-scale (FS) output of the sensor was 74.86 mV and the sensitivity was 2.255 mV/V/kPa. Meanwhile, we applied a pressure ΔP to the sensing diaphragm in steps of 0.1 kPa, and the real-time transient output voltage of the device showed a steady change, as shown in Figure 9b, which demonstrates that the high-pressure sensing resolution allows the device to achieve accurate detection. The piezoresistive pressure sensor had a full-scale (FS) hysteresis error of less than 0.35%FS. A lower repeatability error of 0.37%FS was achieved after five consecutive pressure cycles tested at the same room temperature, as shown in Figure 9c for I to V. The pressure sensor was tested for repeatability for over 300 load and unload cycles at 10 kPa pressure. Figure 9d shows the repeatability results, and the inset shows the voltage variation for seven cycles at 300 and 1500 s each. From the results, we concluded that the sensor performance is very stable, and there is no significant mechanical fatigue.

We reduced the size of the device as much as possible without sacrificing sensitivity by reducing the area and thickness of the diaphragm. However, during processing, residual stresses in amorphous materials such as silicon dioxide and silicon nitride caused deformation of the thin sensing diaphragm and caused nonlinear problems. The prestress in the deformed diaphragm affected the voltage output. The response shown in Figure 8a exhibits a dead band in the range of 0–3 kPa, which is acceptable for some low-pressure monitoring applications.

**Figure 10:**
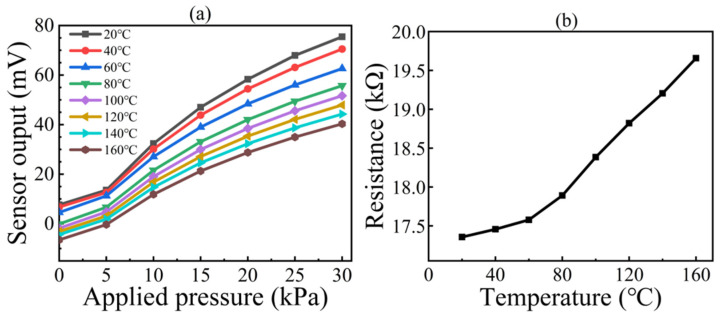
High-temperature characteristics of the sensor: (**a**) output curves at different temperatures; (**b**) resistance–temperature characteristics.

TCO and TCS were used to investigate the variation in the sensor output voltage when pressure was applied at different temperatures. Figure 10a shows the relationship between the applied full-scale pressure and the output at different temperatures. It can be seen that the output voltage decreased as the temperature increased. The sensor had a temperature drift that caused the zero-output voltage as well as the sensitivity to show differences. 

Figure 10b shows the resistance of the device developed in this study, tested over a wide temperature range up to 160 °C. It can be seen that the resistance did not fail and increased as the temperature rose. Based on the change in resistance, the positive temperature coefficient of resistivity (TCR) was calculated to be 0.095% FSS/℃, which indicates the strong high-temperature ability of the device. 

**Figure 11:**
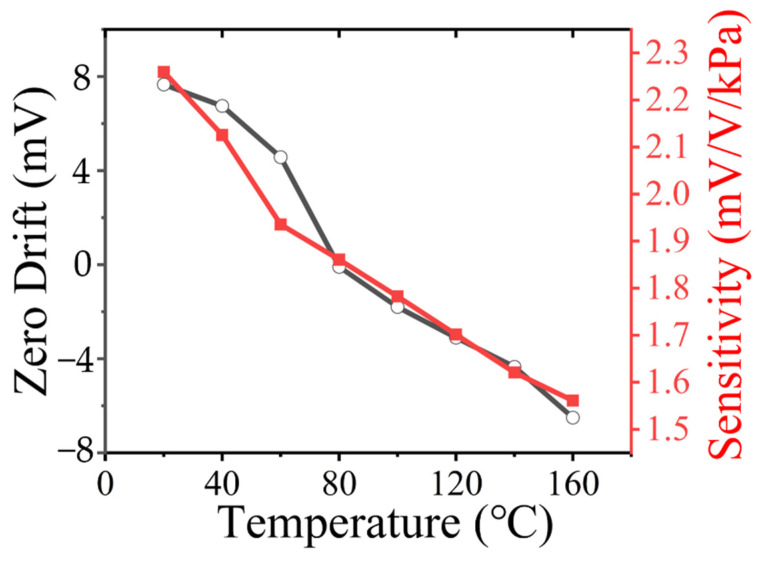
Sensitivity variation and zero output of the sensor at different temperatures.

The zero output and sensitivity varied at different temperatures because the four resistors of the Wheatstone bridge had slightly different doping levels and the presence of residual stresses. As the temperature increased from 20 °C to 160 °C, there was a small decrease in the sensitivity of the sensor as the piezoresistive coefficient decreased with increasing temperature. The sensitivity of the sensor had a temperature coefficient, as shown in Figure 11, with a temperature drift coefficient of −0.221%FS/°C at full scale and a TCO of −0.209%FS/°C.

Finally, our devices have good overall performance. The performance characteristics of the fabricated sensors are listed in Table 3. The measurement performance and size of the fabricated sensors were compared with those of other sensors. Compared with the results of studies of SOI piezoresistive pressure sensors presented in [21,24], the sensitivity of our device is a multiple of their order of magnitude and has the smallest footprint. Compared with the non-SOI silicon-based devices presented in [46], our devices offer good accuracy (high sensitivity, low repeatability, and hysteresis error) combined with a small diaphragm length of 700 μm, so is suitable for low air pressure monitoring applications. Moreover, based on the SOI structure, our device is capable of stable operation in high-temperature environments. The TCO and TCS, as shown in the previous section, show that the device is well-suited for applications at 160 °C and above. Our miniaturized high-temperature sensors have great commercial value. Their performance is comparable to or better than those of commercially available (e.g., Kulite XTE-190 series, MERIT Sensor LP Series, and MEMSensing MSPC04-GDS1) piezoresistive pressure sensors. In addition, our SOI piezoresistive pressure sensors achieve multistage performance with high temperature resistance, high accuracy, and a small footprint. It can provide a technical reference for research on piezoresistive pressure sensors for low air pressure and medium and high temperatures and can meet the needs of devices in consumer electronics, the automotive industry, and medical aviation.

## 5. Conclusions

In this study, piezoresistive MEMS pressure sensors with small size, high sensitivity, and high temperature were fabricated on 8-inch SOI wafers in a standard CMOS-compatible batch process. The stress distribution on the small-sized film at a specific pressure was simulated, and in-depth experiments were performed to determine the device’s performance. The structural layers of the sensor were optimized to improve sensitivity, and the piezoresistive fabrication process was controlled to reduce the temperature drift of the device. The experimental results showed that the sensor had a sensitivity of 2.255 mV/V/kPa and a pressure-sensing resolution of 100 Pa in the range of 0–30 kPa. The full-scale output hysteresis was less than 0.22%FS, and the repeatability error was less than 0.37%FS. The pressure sensor could stably operate at 160 °C with a TCS of −0.221%FS/°C and a TCO of −0.209%FS/°C. The proposed highly sensitive pressure sensor with compact dimensions has potential for use in most consumer electronics and physiological monitoring applications and is more suitable for industrial production and aerospace high-temperature, low-pressure testing areas.

## Figures and Tables

**Table 1:** Stress and strain simulation parameters and results.

Parameter	Value
Diaphragm length (µm)	700
Young’s modulus of Si (GPa)	170
Poisson’s ratio of Si	0.06
Density of Si (kg/m^3^)	2329
Maximum stress (MPa)	250.49
Maximum displacement (µm)	8.06

**Table 2:** Resistance values at different beam currents.

Point Location	2 mA	3 mA	4 mA	5 mA	Unit
Point ①	4731	3098	3895	1818	Ω
Point ②	5283	6760	3548	2427	
Point ③	4088	4059	3880	1991	
Point ④	4719	2411	3855	4349	
Point ⑤	4291	3552	3635	3961	

**Table 3:** Comparisons with a different fabricated sensors.

Parameter	Our Sensor	[21]	[24]	[46]
Structure	SOI	SOI	SOI	SI
Measured pressure range	0–30 kPa	0–140 bar	0–2.5 MPa	0.5–40 kPa
Diaphragm length (µm)	700	750	5000	900
Sensitivity	2.255 mV/V/kPa	0.308 mV/V/bar	0.037 mV/V/kPa	0.328 mV/kPa
Temperature (°C)	20~160	25~200	−40~60	-
TCR (%FS/°C)	0.095	0.364	-	-
Hysteresis (%FS)	0.22	-	-	1.6
Repeatability (%FS)	0.37	-	-	0.63

## Data Availability

The data are available within the article.
